# Effectiveness of telemedicine diabetic retinopathy screening in the USA: a protocol for systematic review and meta-analysis

**DOI:** 10.1186/s13643-023-02201-9

**Published:** 2023-03-16

**Authors:** Tania Padilla Conde, Lauren Robinson, Paras Vora, S. Lee Ware, Arnold Stromberg, Ana Bastos de Carvalho

**Affiliations:** 1 Department of Ophthalmology and Visual Science, University of Kentucky, Lexington, KY USA; 2 Medical Center Library, University of Kentucky, Lexington, KY USA; 3 Department of Statistics, University of Kentucky, Lexington, KY USA

**Keywords:** Telemedicine, Teleophthalmology, Diabetic retinopathy, Diabetic eye disease, Teleretinal screening

## Abstract

**Background:**

Diabetic retinopathy (DR) is the leading cause of vision loss among adults in the USA. Vision loss associated with diabetic retinopathy can be prevented with timely ophthalmologic care, and therefore, it is recommended that individuals with diabetes have annual retinal examinations. There is limited evidence on whether using telemedicine to screen for DR in primary care clinics in the USA effectively leads to increased DR screening rates. The objective of this systematic review is to collate evidence from existing studies to investigate the effectiveness of telemedicine DR screening (TDRS) in primary care clinics on DR screening rates.

**Methods:**

Relevant studies will be identified through searching MEDLINE/PubMed interface, Scopus, and Web of Science from their inception until November 2021, as well as searching reference lists of included studies and previous related review articles or systematic reviews. There will be no restrictions on study design. Eligible studies will include subjects with either type 1 or type 2 diabetes, will evaluate telemedicine technology for screening of DR, will have been conducted in the USA, and will report DR screening rates or data necessary for calculating such rates. Two reviewers will screen search results independently. Risk-of-bias assessment and data extraction will be carried out by two reviewers. The version 2 of the Cochrane risk-of-bias tool (RoB 2) and the Newcastle-Ottawa scale (NOS) tool will be used to assess the quality and validity of individual studies. If feasible, we will conduct random-effects meta-analysis where appropriate. If possible, we will conduct subgroup analyses to explore potential heterogeneity sources (setting, socio-economic status, age, ethnicity, study design, outcomes). We will disseminate the findings through publications and relevant networks.

**Discussion:**

This protocol outlines the methods for systematic review and synthesis of evidence of TDRS and its effect on DR screening rates. The results will be of interest to policy makers and program managers tasked with designing and implementing evidence-based services to prevent and manage diabetes and its complications in similar settings.

**Systematic review registration:**

PROSPERO CRD42021231067.

**Supplementary Information:**

The online version contains supplementary material available at 10.1186/s13643-023-02201-9.

## Background

Diabetic retinopathy (DR) is the leading cause of vision loss among adults in the USA [1]. Modifiable risk factors such as hyperglycemia or hypertension have been implicated in the onset and progression of DR. Sight-threatening complications of diabetic retinopathy such as proliferative diabetic retinopathy (PDR) and diabetic macular edema (DME) can be effectively treated using laser photocoagulation and intravitreal injections. These treatments can reduce the risk of severe vision loss by more than 90% [2]. However, treatment success is dependent on early detection and timely intervention and often requires multiple treatment visits [3].

The American Diabetes Association and the American Academy of Ophthalmology Retina Panel recommend routine dilated eye exams for persons with diabetes, beginning 5 years after diagnosis for type 1 diabetes and at the time of diagnosis and annually (or every 2 years) thereafter for type 2 diabetes [4]. The traditional approach to detecting diabetic eye disease involves a referral to an eye care specialist by the patient’s primary care provider. Screening for diabetic retinopathy with the ultimate goal of enabling access to sight-saving treatment has been shown to be both clinically effective and cost-effective [3]. However, fewer than half of all diabetic patients in the USA receive the recommended annual screening for diabetic retinopathy [5]. Socioeconomic and geographic barriers to care, delayed referrals from PCPs, and lack of patient education have been cited as reasons for low rates of screening for diabetic retinopathy [6].

Telemedicine with retinal imaging and remote interpretation by an expert has been shown to be a promising and efficacious technology designed to identify patients with diabetic retinopathy [7]. Retinal images are acquired via non-mydriatic cameras that can be operated with minimal instruction and are sent for remote evaluation by an ophthalmologist or trained fundus grader. Referral is then placed to an ophthalmologist if treatment is indicated based on the Early Treatment Diabetic Retinopathy Study (ETDRS) definition of moderate nonproliferative diabetic retinopathy or any sign of diabetic maculopathy [8]. Remote imaging to detect possible vision-threatening pathology in patients who may otherwise be asymptomatic may therefore reduce many barriers such as transportation, concern with pupillary dilation, and poor adherence to recommended eye exams [9].

Previous literature reviews and studies have indicated that telemedicine diabetic retinopathy screening (TDRS) has a high overall diagnostic accuracy, with sensitivity exceeding 80% and specificity of 90% or greater [10]. Within the Veterans Administration health system, telemedicine diabetic retinopathy screening was found to be cost-effective [11], and countries such as the UK have demonstrated successful country-wide public screening programs for diabetic retinopathy using telemedicine [12].

In the USA, establishing such state- or country-wide screening programs ubiquitously may prove more complex, due to inherent differences in health systems, as well as fragmentation of healthcare payers. Therefore, we have seen mostly smaller-scale screening networks [13], and single-site primary care clinics adopt TDRS, with the goal of reaching underserved populations in remote, rural, or urban settings and improving early detection of diabetic retinopathy [13–15]. TDRS can be cost-effective [16] if screening is done in populations with a null baseline screening rate [17] or if TDRS rates reach at least 65% [18]. However, TDRS implementation and uptake can be fraught with barriers [19–21], and it is possible that such significant increase in rates, as is required for cost-efficacy, may not be attainable. Therefore, it is essential to determine the effectiveness of TDRS in increasing DR screening rates in real-world conditions. To date, no systematic reviews or meta-analyses have addressed this issue.

### Objective

The aim of this systematic review is to assess effectiveness of TDRS on improving DR screening rates in primary care clinics in the USA.

### Intervention

The intervention is TDRS, defined as digital retinal imaging acquired via a fundus camera and sent for remote evaluation and diagnosis of diabetic retinopathy by a trained fundus picture reader (ophthalmologist or trained fundus grader).

## Methods

The protocol is being reported in accordance with the reporting guidelines of the Preferred Reporting Items for Systematic Reviews and Meta-Analyses Protocols (PRISMA-P) statement [22] (see checklist in Additional file 1). The study protocol is also registered in the International Prospective Register of Systematic Reviews (PROSPERO, Registration ID: CRD42021231067).

### Study eligibility requirements

Eligible studies will include peer-reviewed publications of studies conducted in the USA in primary care clinics of any setting (community clinics, facility based, rural, urban), which examine the rates of DR screening pre- and post-initiation of a TDRS intervention. Studies reporting a comparator (defined as rates of screening in primary care clinics without TDRS intervention or before its initiation) will also be eligible. We will restrict to publications in the English language. We will only include studies where a full-text report is available. Studies will be excluded if missing full text when this is not accessible through either EndNote search or manual search with institutional resources. Eligible population will include adults (aged ≥ 18 years) with type 1 or type 2 diabetes who have been identified as eligible for TDRS in primary care clinics in the USA. Exclusion criteria include pregnant women and individuals with previously diagnosed diabetic retinopathy. We will include any study reporting data that allows computation of at least one primary outcome (Table 1).

**Table 1:** Comparator and outcomes of interest

Comparator	Rates of screening in primary care clinics without TDRS intervention or before its initiation
**Outcomes**	Primary
	1. Proportion of patients receiving diabetic retinopathy screening (through either exam with eye care provider or via telemedicine) among those for whom it is recommended
	2. Proportion of patients receiving telemedicine diabetic retinopathy screening among those to whom it is recommended
	Secondary
	3. Proportion of patients with gradable images on telemedicine diabetic retinopathy screening
	4. Proportion of patients completing referral to eye care specialist after a positive screening test
	5. Proportion of patients completing timely recommended referral to eye care specialist after a positive screening test

### Search strategy

The comprehensive search strategy was developed by the research team, which includes ophthalmologists, a medical librarian, a physician with public health expertise, and a statistician. Relevant studies will be identified through searching MEDLINE PubMed interface, Scopus (Elsevier), and Web of Science Core Collection (Clarivate) [23–25]. The coverages of each database are described in Table 2. The researchers selected these databases based on institutional availability, alongside the breadth of discipline coverage. These sources will be searched using a combination of relevant search terms that were developed through an initial scan of the literature using MEDLINE PubMed. The search terms were adapted to match the syntax of each electronic database. Subject headings were used in appropriate databases. To ensure this systematic review captures the full extent of the literature, extensive hand-searching of reference lists will be completed on all included studies.

**Table 2:** Database access and coverage

Database	Vendor	Coverage start
MEDLINE via PubMed	NIH	1966
Web of Science Core Collection	Clarivate	Sciences: 1900-present Social sciences: 1900-present Arts & humanities: 1975-present
Scopus	Elsevier	1996

The final MEDLINE PubMed search strategy was developed using a MeSH analysis of 11 sentinel articles [26]. These sentinel articles were also used throughout development to test the reliability of the search string. The final search string is as follows: ("Diabetic Retinopathy"[Mesh] OR "Diabetic Retinopath*"[tiab] OR "diabetic eye disease*"[tiab] OR "diabetic eye examin*"[tiab]) OR (("Diabetes Mellitus"[Mesh] OR "Diabetes Mellitus, Type 2"[Mesh] OR "Diabetes Mellitus, Type 1"[Mesh] OR "Diabetes Complications"[Mesh] OR diabetes*[tiab] OR T2DM[tiab] OR MODY[tiab] OR "Type 1"[tiab] OR "Type 2"[tiab] OR "Type I"[tiab] OR "Type II"[tiab] OR NIDDM[tiab] OR "Autoimmune Diabetes"[tiab]) AND ("Retina"[Mesh] OR "Macular Edema"[Mesh] OR "retinal screen*"[tiab] OR "retina*"[tiab] OR "Macular Edema*"[tiab])) AND ((("Telemedicine"[Mesh] OR "Remote Consultation"[Mesh]) OR (Telemedicine[tiab] OR "Mobile Health"[tiab] OR mHealth[tiab] OR eHealth[tiab] OR Telehealth[tiab] Teleophthalmology[tiab] OR Telecare[tiab] OR Teleconsult*[tiab] OR "tele medicine"[tiab] OR telemed[tiab] OR telemedical[tiab] OR "tele care"[tiab] OR "tele health"[tiab] OR "tele consult*"[tiab] OR "tele medical"[tiab] OR "Teleretinal screening" OR "retinal imaging technolog*"[tiab])) OR ((remote[tiab] OR virtual[tiab]) AND (evaluation*[tiab] OR consult*[tiab] OR management[tiab] OR assessment*[tiab]))).

### Search strategy data management

The medical librarian will create a table recording essential information, including but not limited to date searches that were conducted, search strategies, and number of search results. The resulting citations will be exported to EndNote citation management software. The groups function will be used to sort references by database. Since the group’s function keeps a record of citations, this process will act as a backup to the table mentioned previously. The EndNote library will then be uploaded to Covidence [27] for deduplication. After the initial title/abstract review has been completed by the researchers, we will use EndNote and all available institutional collections and resources to find full-text articles. Full texts will then be uploaded to Covidence for full-text review. The medical librarian will also maintain records of the exported RIS files from the 3 databases searched, the original MeSH analysis, and other essential information. To ensure continuous access and backup, we will keep remote copies of all files using the institution’s Microsoft OneDrive.

### Quality assessment

The version 2 of the Cochrane risk-of-bias tool (RoB 2) will be used to assess the risk of bias of randomized controlled trials (RCTs) [28]. The Newcastle-Ottawa scale (NOS) tool will be used to assess the methodological quality of nonrandomized studies of interventions [29]. Two reviewers will independently assess the quality of all studies. Any differences of opinion regarding quality will be resolved by discussion, or a third reviewer will arbitrate if a consensus is not reached.

### Selection process

Following de-duplication, two reviewers will screen the titles and abstracts of all the references generated to determine if the complete manuscript should be retrieved. Any discrepancies will be resolved by discussion, or a third reviewer will arbitrate if a consensus is not reached. Potentially eligible studies identified in the first phase of screening will then be screened for inclusion against the eligibility criteria at the full-text level by two reviewers. Any differences of opinion will be resolved by discussion, or a third reviewer will arbitrate if a consensus is not reached. Reasons for exclusion will be recorded. The number of records identified, retrieved, screened, assessed, included, and excluded in the review, and reasons for exclusions, will be summarized in a PRISMA flow diagram (version 2020).

### Data collection process

Once the eligible studies have been identified, data will be extracted by one reviewer using the Covidence software. Extracted data will be checked by a second reviewer. Any disagreements will be resolved by discussion until consensus is reached. Relevant data to be collected include the following: study identification with year of publication, primary study aim, study setting, study population, study design, demographics and baseline characteristics of participants, details of intervention, study outcomes (Table 1), and study limitations. If a study does not have all relevant data to be collected, the authors will contact the corresponding author to inquire about potential unpublished data.

### Strategy for data analysis and synthesis

We anticipate that there will be clinical and methodological diversity in the studies that we find. We plan to summarize the data narratively, regardless of whether we are able to do a meta-analysis, following SWiM reporting guidance: synthesis without meta-analysis [30].

We will obtain sample sizes, variance estimates, and confidence intervals from our primary outcomes (Table 1) from each publication. We will report these outcomes in terms of proportions in intervention (TDRS) and comparator groups using confidence intervals for proportions. Secondary outcomes will be compared using confidence intervals for proportions as well. When possible, we will calculate the prevalence and/or incidence risk ratios with 95% confidence intervals to provide further insights in addition to differences in proportions. However, this will vary depending on how data are reported by studies. If sufficient data are available, we will calculate *I*^2^, a measure of the percentage of variation across studies that is due to heterogeneity rather than chance. Heterogeneity will be assessed by examining study design, geographical location, demographic characteristics, nature of the interventions, and study setting. To assess the certainty of the synthesis findings, we will use the systematic GRADE approach adapted for narratively summarized results [31].

For the publications that have enough information about the variability of each estimate, we will use random effects statistical models to combine data from each study and perform a meta-analysis. Resampling methods, such as the bootstrap, will be considered for combining rates across studies. Both random-effect models and bootstrapping are incorporated into the metafor R package. Analysis will be done using SAS or R software as appropriate [32].

### Analysis of subgroups or subsets

Data allowing, we will group studies by setting, socio-economic status, age, ethnicity, study design, and outcomes. For each comparison and outcome, we will provide a description of the findings alongside the certainty of the evidence, ensuring consistency with the review question and providing a judgment as to the extent to which the studies contribute to the synthesis.

## Discussion

This systematic review will identify and synthesize the published evidence on effectiveness of TDRS in increasing DR screening rates in primary care clinics in the USA. There are published systematic reviews looking at cost-effectiveness and accuracy of TDRS; however, this will be the first systematic review looking at the effect of TDRS on screening rates in US primary care clinics.

The information gained from this review will support program implementers and inform public health policies, guidelines, and practices around the use of TDRS in primary care clinics. This review may also identify areas for improvement in screening for diabetic retinopathy not made explicit in previous studies. As telemedicine becomes more widely used and available both in the USA and internationally, this study will enlighten the current status of TDRS and may prove useful for other countries implementing TDRS in primary care clinics systems or at a national level.

### Limitations

High heterogeneity between included studies might arise from differences in study characteristics not anticipated by authors or not explained at the study level. Keyword searches may fail to turn up related materials that do not specifically use the search term (false negatives). Excluding studies with no full text accessible may limit the number of studies included. This could increase risk of bias on some outcome variables and inconclusive results. Reporting bias may be encountered, as studies may present positive findings and may not fully report all data.

### Protocol amendments

Any amendments to this protocol while carrying out the systematic review will be documented and reported in both the PROSPERO register and any subsequent publications.

### Dissemination plans

The findings of this systematic review will be disseminated through publication in scientific medical journals targeting eye care, diabetes, and systematic reviews/meta-analysis and in scientific conferences addressing these health issues. In addition, the results will also be shared with potential stakeholders, which may include primary healthcare systems in rural or urban poor settings in the USA, the Department for Public Health in Kentucky, and other states with large proportions of rural populations, and stakeholders of state and federal healthcare programs in the USA (Medicare and Medicaid).

## Supplementary Information

**Additional file 1.** PRISMA 2020 Checklist.

## Data Availability

Not applicable.

## Abbreviations

DR: Diabetic retinopathy
DME: Diabetic macular edema
ETDRS: Early Treatment Diabetic Retinopathy Study
PRISMA-P: Preferred Reporting Items for Systematic Reviews and Meta-Analyses Protocols
PDR: Proliferative diabetic retinopathy
QUADAS-2: Quality Assessment of Diagnostic Accuracy Study 2
TDRS: Telemedicine diabetic retinopathy screening
